# Chemokines, molecular drivers of thromboinflammation and immunothrombosis

**DOI:** 10.3389/fimmu.2023.1276353

**Published:** 2023-10-26

**Authors:** Julian Leberzammer, Philipp von Hundelshausen

**Affiliations:** ^1^ Institute of Cardiovascular Regeneration, Goethe University Frankfurt, Frankfurt, Germany; ^2^ Department of Cardiology and Angiology, Goethe University Frankfurt, University Hospital, Frankfurt, Germany; ^3^ German Center for Cardiovascular Research (DZHK), Partner Site Munich Heart Alliance, Munich, Germany; ^4^ Institute for Cardiovascular Prevention, Institut für Prophylaxe und Epidemiologie der Kreislaufkrankheiten (IPEK), Ludwig-Maximilians-Universität München, Munich, Germany

**Keywords:** platelet, atherothrombosis, inflammation, leukocyte, red blood cell, endothelial cell

## Abstract

Blood clotting is a finely regulated process that is essential for hemostasis. However, when dysregulated or spontaneous, it promotes thrombotic disorders. The fact that these are triggered, accompanied and amplified by inflammation is reflected in the term thromboinflammation that includes chemokines. The role of chemokines in thrombosis is therefore illuminated from a cellular perspective, where endothelial cells, platelets, red blood cells, and leukocytes may be both the source and target of chemokines. Chemokine-dependent prothrombotic processes may thereby occur independently of chemokine receptors or be mediated by chemokine receptors, although the binding and activation of classical G protein-coupled receptors and their signaling pathways differ from those of atypical chemokine receptors, which do not function via cell activation and recruitment. Regardless of binding to their receptors, chemokines can induce thrombosis by forming platelet-activating immune complexes with heparin or other polyanions that are pathognomonic for HIT and VITT. In addition, chemokines can bind to NETs and alter their structure. They also change the electrical charge of the cell surface of platelets and interact with coagulation factors, thereby modulating the balance of fibrinolysis and coagulation. Moreover, CXCL12 activates CXCR4 on platelets independently of classical migratory chemokine activity and causes aggregation and thrombosis via the PI3Kβ and Btk signaling pathways. In contrast, typical chemokine-chemokine receptor interactions are involved in the processes that contribute to the adhesiveness of the endothelium in the initial phase of venous thrombosis, where neutrophils and monocytes subsequently accumulate in massive numbers. Later, the reorganization and resolution of a thrombus require coordinated cell migration and invasion of the thrombus, and, as such, indeed, chemokines recruit leukocytes to existing thrombi. Therefore, chemokines contribute in many independent ways to thrombosis.

## 1 Introduction

Inflammation and thrombosis are closely intertwined which is long known for atherosclerotic cardiovascular disease (1). Insight into this close interplay between the immune and hemostatic systems, coined the term immunothrombosis (2). This term refers to a primarily beneficial mechanism of how the organism can contain a noxious threat, for example, from a microbial invasion by preventing a further spread through the blood. Uncontrolled aberrant immunothrombosis wreaks havoc, causing thromboinflammation (3). In this way, immunothrombosis and thromboinflammation are two sides of the same coin. Among many relevant inflammatory effector molecules are chemokines that play an important role in inflammation, atherosclerosis and thrombosis and link in several aspects the immune and hemostatic system. They are a well-described group of chemotactic mediators of about 50 members. Despite a large sequence dissimilarity, chemokines display a typical molecular fold that resembles a Greek key. A structure held together by two cysteines that follow a flexible N-terminal tail and form bridges to loops around residue positions 30 (30s loop) and 50 (50s loop). The cysteine motif, the namesake of the CC and CXC family, is followed by the N-loop, which transitions into a β-sheet of three β-strands linked to the C-terminal helix. Exceptions are CX3CL1 which is the only chemokine that contains Nt cysteines separated by three amino acids, and like CXCL16, is *a priori* not soluble but integrated into the plasma membrane.

Most of the chemokines have one or more high-affinity receptors that belong to the 7TM receptor family that typically signal through G-proteins, mostly Gαi, GRKs or independent of G-proteins through arrestins or act as atypical receptors without signaling functioning as chemokine sink.

## 2 The endothelium and vascular wall are both a source and target of chemokines in thrombosis

The endothelial layer passively protects against thrombosis by separating the blood from the pro-thrombotic components of the underlying vascular wall, such as matrix proteins, or atherosclerotic plaque material including collagens. Denudation or dysfunction by injury, virus-induced endothelitis or plaque rupture exposes these structures (4). Healing requires migration and proliferation of endothelial cells (5). The endothelium can also play an active role in promoting thrombosis by supporting the extrinsic coagulation cascade and the rolling and adhesion of platelets. Although several chemokines and their receptors are expressed by endothelial cells, and their ability to control cell migration and induce cell adhesion is essential for thrombosis, the experimental knowledge about chemokine-driven endothelial-specific processes in thrombosis and healing is relatively limited.

Under steady-state conditions, single-cell RNAseq (tabula muris, tabula sapiens) reveals substantial transcripts of atypical chemokine receptors ACKR1/DARC >ACKR3/CXCR7 > ACKR2/D6 in endothelial cells from multiple organs and vascular beds, followed by classical chemokine receptors CXCR4, CCR10/GPR2 (**Table 1**). At lower levels, CCR1, CCR2, CCR5 and CX3CR1 are not detectable, which does not exclude a functional presence of other chemokine receptors in endothelial cells. Especially after endothelial activation, *de novo* transcription and a change in expression pattern will occur (7, 8). This renders endothelial cells susceptible to binding and activation by a large variety of chemokines.

**Table 1:** Chemokine receptor expression by human endothelial cells.

Vascular bed	Readout	Total cells	CCR1	CCR2	CCR3	CCR4	CCR5	CCR6	CCR7	CCR8	CCR9	CCR10	CX3CR1	XCR1
**Capillary EC**	**log2cpm**	515 ± 534	0.0 ± 0.0	0.0 ± 0.0	0.0 ± 0.0	0.0 ± 0.0	0.0 ± 0.0	0.0 ± 0.0	0.0 ± 0.0	0.0 ± 0.0	0.0 ± 0.0	0.1 ± 0.1	0.0 ± 0.0	0.0 ± 0.0
	**%pos. cells**		0.0 ± 0.1	0.0 ± 0.1	0.0 ± 0.0	0.0 ± 0.0	0.1 ± 0.2	0.0 ± 0.0	0.2 ± 0.4	0.0 ± 0.0	0.0 ± 0.0	2.4 ± 2.6	0.2 ± 0.5	0.0 ± 0.0
**Vein EC**	**log2cpm**	564 ± 702	0.0 ± 0.0	0.0 ± 0.0	0.0 ± 0.0	0.0 ± 0.0	0.0 ± 0.0	0.0 ± 0.0	0.0 ± 0.0	0.0 ± 0.0	0.0 ± 0.0	0.1 ± 0.1	0.0 ± 0.0	0.0 ± 0.0
	**% pos. cells**		0.3 ± 0.5	0.2 ± 0.3	0.0 ± 0.0	0.3 ± 0.6	0.1 ± 0.3	0.0 ± 0.0	0.4 ± 0.5	0.0 ± 0.0	0.0 ± 0.0	1.2 ± 0.8	0.3 ± 0.7	0.0 ± 0.1
**Lymph EC**	**log2cpm**	84 ± 85	0.0 ± 0.0	0.0 ± 0.0	0.0 ± 0.0	0.0 ± 0.0	0.0 ± 0.0	0.0 ± 0.0	0.0 ± 0.0	0.0 ± 0.0	0.0 ± 0.0	0.1 ± 0.2	0.0 ± 0.0	0.0 ± 0.0
	**% pos. cells**		0.3 ± 1.0	0.2 ± 0.4	0.1 ± 0.5	0.0 ± 0.0	0.2 ± 0.4	0.1 ± 0.3	0.3 ± 0.6	0.0 ± 0.0	0.0 ± 0.0	2.4 ± 3.2	0.2 ± 0.5	0.0 ± 0.1
**Artery EC**	**log2cpm**	249 ± 436	0.0 ± 0.0	0.0 ± 0.0	0.0 ± 0.0	0.0 ± 0.0	0.0 ± 0.0	0.0 ± 0.0	0.0 ± 0.0	0.0 ± 0.0	0.0 ± 0.0	0.1 ± 0.1	0.0 ± 0.0	0.0 ± 0.0
	**%pos. cells**		0.5 ± 0.3	0.3 ± 0.2	0.0 ± 0.1	0.6 ± 0.0	0.3 ± 0.2	0.0 ± 0.1	0.5 ± 0.3	0.0 ± 0.0	0.0 ± 0.0	0.8 ± 2.4	0.7 ± 0.2	0.1 ± 0.0
Vascular bed	Readout	Total cells	ACKR1	ACKR2	ACKR3	ACKR4	GPR 182	CXCR1	CXCR2	CXCR3	CXCR4	CXCR5	CXCR6	CXCR8
**Capillary EC**	**log2cpm**	515 ± 534	1.9 ± 2.7	0.0 ± 0.1	0.1 ± 0.1	0.1 ± 0.1	0.0 ± 0.0	0.0 ± 0.0	0.0 ± 0.0	0.0 ± 0.0	0.1 ± 0.1	0.0 ± 0.0	0.0 ± 0.0	0.0 ± 0.0
	**%pos. cells**		30 ± 44	0.2 ± 0.5	7.2 ± 8.0	1.1 ± 1.1	0.0 ± 0.0	0.0 ± 0.0	0.2 ± 0.3	0.0 ± 0.0	8.0 ± 8.1	0.4 ± 0.7	0.0 ± 0.0	0.7 ± 0.7
**Vein EC**	**log2cpm**	564 ± 702	4.7 ± 1.0	0.1 ± 0.0	0.7 ± 0.6	0.2 ± 0.1	0.0 ± 0.0	0.0 ± 0.0	0.0 ± 0.0	0.0 ± 0.0	0.1 ± 0.1	0.0 ± 0.0	0.0 ± 0.0	0.0 ± 0.0
	**% pos. cells**		82 ± 15	1.1 ± 0.6	30 ± 20	3.0 ± 1.5	0.3 ± 0.4	0.2 ± 0.3	0.6 ± 0.5	0.1 ± 0.1	7.6 ± 9.6	0.1 ± 0.1	0.2 ± 0.3	0.4 ± 0.3
**Lymph EC**	**log2cpm**	84 ± 85	0.8 ± 1.1	1.3 ± 1.1	0.9 ± 0.7	0.7 ± 1.4	1.7 ± 1.5	0.0 ± 0.0	0.0 ± 0.0	0.0 ± 0.0	0.1 ± 0.1	0.0 ± 0.0	0.0 ± 0.0	0.0 ± 0.0
	**%pos. cells**		20 ± 23	19 ± 14	39 ± 24	8.0 ± 15.2	20 ± 16	0.0 ± 0.0	0.1 ± 0.4	0.4 ± 1.0	7.6 ± 10.9	0.1 ± 0.4	0.1 ± 0.3	0.0 ± 0.2
**Artery EC**	**log2cpm**	249 ± 436	1.0 ± 0.8	0.0 ± 1.3	0.6 ± 0.9	0.1 ± 0.7	0.0 ± 0.0	0.0 ± 0.0	0.0 ± 0.0	0.0 ± 0.0	0.1 ± 0.1	0.0 ± 0.0	0.0 ± 0.0	0.0 ± 0.0
	**%pos. cells**		15 ± 20	0.6 ± 18.6	20 ± 39	1.5 ± 8.0	0.0 ± 0.0	0.3 ± 0.0	0.5 ± 0.1	0.1 ± 0.4	9.6 ± 7.6	0.1 ± 0.1	0.3 ± 0.1	0.3 ± 0.0

Gene expression of chemokine receptors from human endothelial cells extracted from tabula sapiens data (6), shown as mean ± SEM log2cmp and percent positive cells (6). Only data from endothelial cells assigned to a specific vascular bed are shown. The color intensity corresponds to the degree of expression. ACKR3=CXCR7, GPR182 has been tentatively named ACKR5 and CXCR8= GPR35.

**Figure 1:**
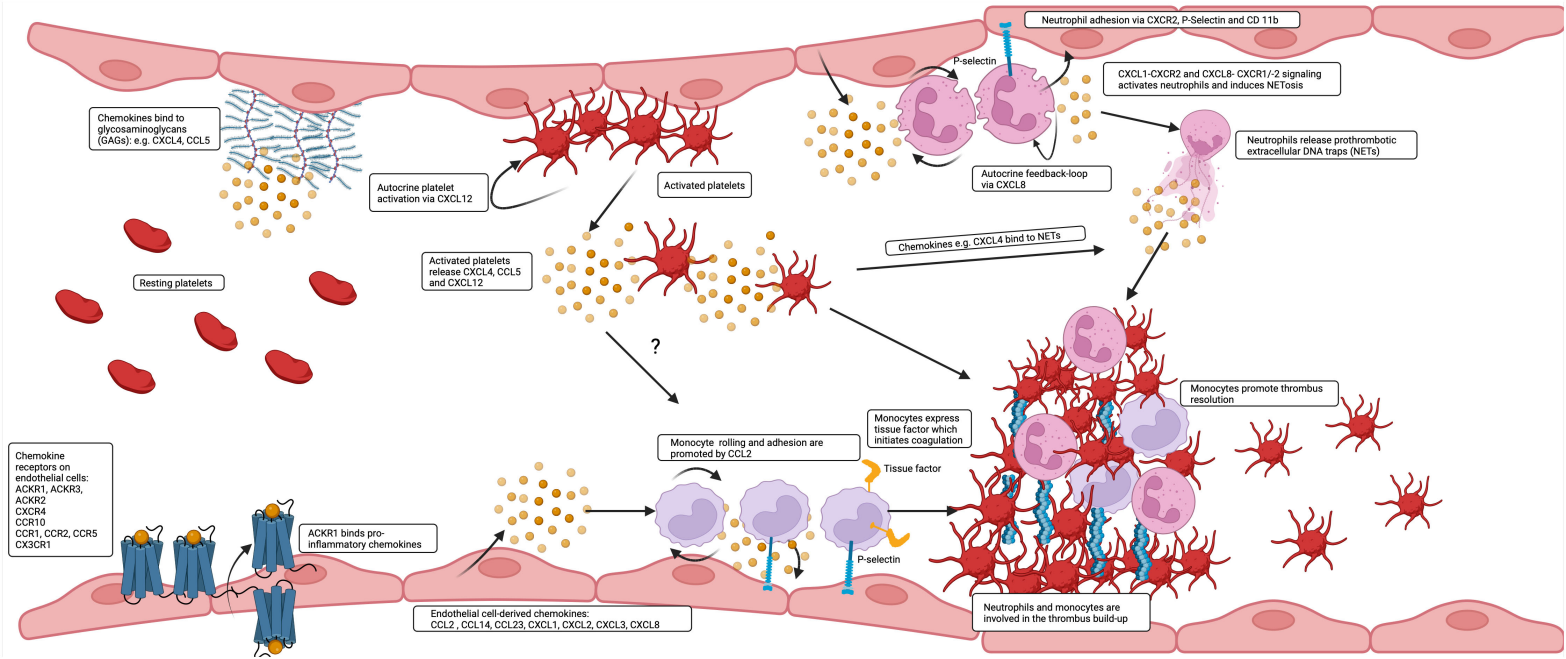
Chemokines are produced by and bind to endothelial cells to affect thrombus build up and resolution. A great variety of chemokines is presented by the endothelium. Inflammatory chemokines are transported and presented by ACKR1 to the luminal surface or are released and deposited by platelets and leukocytes or directly released by endothelial cells and additionally presented by GAGs. CCL2 and CXCL8 contribute in interaction with tissue factor (TF) and P-selectin to the prothrombotic accumulation of monocytes and neutrophils on the endothelium and are at later stages also involved in thrombus resolution.

ACKR1/DARC is preferentially expressed under steady-state conditions by vein and capillary endothelial cells (**Table 1**) but is upregulated under inflammatory conditions and may play a role for arteries through its expression in the vasa vasorum (9, 10). ACKR1 mainly binds inflammatory chemokines of the CC and CXC families, such as CCL2, CXCL1, and CXCL8, and actively transports them from the tissue through the body of an endothelial cell (transcytosis) to present them at the surface (**Figure 1**) (11, 12). ACKR1 binds also CXCL12 which is regarded as a homeostatic chemokine (13). Recent evidence indicates that ACKR1/DARC preferentially binds dimeric CXCL12 over monomeric CXCL12, presumably modulating CXCL12 activity by scavenging at higher CXCL12 concentrations when dimers dominate (14). Chemokine presentation be endothelial cells could be an important step in initiating thrombosis, in which the accumulation of myeloid cells is a major factor. Conversely, chemokines can be transported from the vascular lumen into the endothelial cell. This is true for CCL5 and CXCL4 (platelet factor 4, PF4), which are rapidly transported intracellularly to the nucleus of venous endothelial cells by active endocytosis that requires GPCR signaling (15). The effects of this process on cell recruitment and endothelial cell function could also influence thrombotic processes, which remains to be shown (15).

CXCL1/CXCL2 and CCL2 activate CXCR1/CXCR2 and CCR2, respectively. These chemokines are important for thrombus resolution by organizing monocyte infiltration into the venous thrombus, which results in a reduction in thrombus size and collagen content over the course of three weeks (16, 17). CCL2 is expressed by arterial, venous and capillary endothelial cells (6). The physiological relevance of this expression in the human system is supported by the finding that tissue factor (TF) drives endothelial CCL2 expression, leading to smooth muscle cell migration toward endothelial cells and thus facilitating the maturation of newly formed microvessels (18). In the time course of venous thrombosis simulated in a mouse model, CCL2 levels increase in the vein wall and, to a lesser extent, in the thrombus itself by day seven. Direct injection of CCL2 into a developing ligature-induced thrombus increases the number of monocytes in the vein and decreases the size of the thrombus after one week (19). It is conceivable that systemic administration of CCL2 or local application of chemokine-loaded hydrogels (20) could be beneficial and help close endothelial gaps after injury by promoting CCR2-mediated EC migration. However, an increase in circulating CCL2 could lead to adverse effects due to increased mobilization of monocytes from the bone marrow (21).

ACKR3/CXCR7, a receptor for CXCL12, CXCL11, and also opioid peptides, is expressed by arterial and venous endothelial cells (**Table 1**). Recently, by using molecular reporters for ACKR3, ACKR4, and GPR182, the expression of these receptors in the endothelial system could be visualized and mapped (22). GPR182 corresponds to an endothelial atypical chemokine receptor that binds CXCL9, CXCL10, CXCL12, and CXCL13 (23, 24). Because ACKR4 and GPR182 are expressed predominantly by sinusoids and in endothelial cells of lymphatic organs, their expression pattern argues against a direct role in arterial and venous thrombosis. Nevertheless, a role in immunothrombosis to contain infection can be envisioned. When CXCR4 and ACKR3 are co-expressed, such as in endothelial cells, they associate to heteromers at the membrane level, and interact via activation of CXCR4 and downstream GPCR kinases (GRK) at the signaling level. The activation of GRK2 by CXCR4 leads to the phosphorylation of ACKR3, which in turn promotes the internalization and degradation of CXCL12 (25). This was shown in HEK transfectants, and it will be important to see how these mechanisms translate into the vascular environment where CXCR4 and ACKR3 are coexpressed by endothelial cells.

The CXCL12-CXCR4 pathway is essential, and the deficiency of either gene results in fatal embryonic vasculature malformations. CXCR4 is expressed by two types of endothelial cells: TIP cells, which are specialized for angiogenesis, and arterial endothelial cells where it protects against atherosclerosis by strengthening VE-cadherin VE-PTP interactions to maintain vessel integrity (26). Endothelium-specific effects of CXCR4 in *in vivo* thrombosis models have yet to be tested. However, a study has shown that a prevalent CXCL12 gene polymorphism linked to heightened CXCL12 production predisposes patients with chronic myeloproliferative disorders to arterial thrombosis, potentially due to the influence of VEGF (27). A prothrombotic role for the CXCL12/CXCR4/ACKR3 axis has also been proposed for the thrombotic microangiopathy caused by bacterial infections with Shiga toxin-producing E. coli that leads to the hemolytic uremic syndrome (HUS) (28). In this scenario, the exotoxin alters the endothelial phenotype and is implicated in the pathogenesis of microangiopathy in mouse models. The exotoxin stimulates the gene and protein expression of CXCL12, as well as that of its receptors CXCR4 and ACKR3, mimicking the conditions observed in children afflicted with E.coli O157:H7 who further develop HUS where CXCL12 levels are similarly elevated. In the animal model, blocking the CXCL12-CXCR4 pathway with AMD3100 prevents activation of endothelial cells, decreases permeability of endothelium, and enhances survival (28). The impact of the CXCL12-CXCR4 axis on endothelial permeability appears to vary according to the specific type of endothelial cells. In HMVEC and the microvasculature of the kidney and brain, activation of CXCR4 results in increased endothelial permeability, while in arterial endothelial cells of the aorta CXCR4 is crucial for maintaining vascular integrity (26, 29). A weakening of the endothelial barrier will predispose to plaque rupture and arterial thrombosis. In this respect, the increased expression of CXCL12 in unstable atherosclerotic plaques may play a role (30). Moreover, the CXCL12-CXCR4 axis also drives arterial thrombosis by inducing platelet activation (31). Therefore, targeting CXCL12-CXCR4 may prevent and impede thrombosis, although it is crucial to take into account the possible negative impacts on atherogenesis and bone marrow homeostasis. Healing following endothelial injury is a significant predisposing factor for thrombosis. The ability to restore the integrity of the vascular lining is enhanced by factors such as the release of CXCL12 from platelets, apoptotic vascular smooth muscle cells, and the presence of CXCR4 on circulating endothelial progenitor cells. These factors promote EPC recruitment and induce endothelial cell migration (32–34).

Laminar shear stress affects the expression of chemokines and chemokine receptors. For instance, high shear stress leads to a decrease in the expression of the CXCR4 gene and protein in endothelial cells (35). Therefore, investigating endothelial cell-specific knockouts of chemokine receptors in models of arterial thrombosis, venous thrombosis and thrombus resolution are required to understand the role of endothelial chemokine receptors as targets to prevent and treat thromboinflammation.

Under steady-state conditions, arterial, venous and capillary endothelial cells express the transmembrane chemokine CX3CL1 (fractalkine), which is upregulated in endothelial dysfunction. It contributes to the activation of platelets via its receptor CX3CR1 whereas platelet accumulation is mediated via a VWF-GPIb-dependent mechanism, independent its receptor CX3CR1 (36, 37). This mechanism of platelet adhesion has been associated with atherogenesis and likewise may play a role in platelet accumulation in thrombosis (36, 37). Additionally, the accumulation of myeloid cells has been identified as a crucial factor for thrombosis in mouse models, aside from platelet adhesion. One hour after the onset of flow restriction in a mouse model of venous thrombosis induced by blood stasis in the vena cava, monocytes and neutrophils begin to cover the intact endothelial surface. These leukocytes are crucially involved in the build-up and composition of the thrombus (38). Restricting venous flow increased levels of inflammatory molecules including IL-6 and the chemokines CXCL1, CXCL5, and CCL2, leading to inflammatory cell recruitment. The origin and precise role of these chemokines for this process and subsequent steps of thrombus formation and resolution remain to be elucidated (38).

Gene expression of CCL14, CCL23, CXCL1, CXCL2, CXCL3, and CXCL8 is robustly detectable in vascular endothelial cells, reflecting constitutive transcription. In contrast, other chemokine genes such as CCL5 and CXCL10 are barely expressed at rest but inducible by TNF-α activation (39).

It has been shown that chemokine secretion from human endothelial cells (HUVEC) originates from distinct granules. CXCL8 is stored in Weibel-Palade bodies, whereas CCL2, CXCL2 and CCL26 are kept in smaller vesicles released by secretagogues such as histamine, whereas CXCL10 and CCL5 when expressed are not sensitive to secretagogues (40).

Chemokines are avid binders of glycosaminoglycans (GAG), the linear sugar chains of proteoglycans that form a large part of the endothelial glycocalyx (41). GAG of the glycocalyx generate a scaffold and sink for chemokines so that an equilibrium of immobile and soluble chemokines forms a cloud around endothelial cells, which explains why GAG-chemokine interactions are essential for their function. Yet, in many instances, GAG binding competes with receptor binding (42). Chemokines can be released from circulating cells including platelets and deposited on the endothelial surface/glycocalyx. Examples are CCL5 and CXCL4, which are released from activated platelets or by their microparticles and bind to endothelial cells to subsequently activate and recruit leukocytes (43–45). CCL5, immobilized on endothelial cells, forms higher oligomeric structures that appear as strings and are shear flow resistant (46). The binding of chemokines to GAG and proteoglycans can occur not only on the endothelial glycocalyx, but proteoglycans are also constituents of eroded plaques that are substrates for coronary thrombosis (47).

Therefore, a multitude of chemokines is expressed in healthy veins by cells of the vascular wall and the endothelium. Chemokines are upregulated by flow disturbance, be it stasis or shear gradients in the nascent thrombus. Upregulation reflects inflammation and endothelial cell activation, which are the initial steps in deep vein thrombosis and likely also in microvascular thrombosis, where leukocyte adhesion is responsible for the ensuing steps of platelet aggregation, fibrin formation and thrombus build-up (**Figure 1**).

### 2.1 Chemokines affect coagulation through interaction with endothelial GAGs, plasma proteins and NETs

Soluble glycosaminoglycans (GAGs) are highly effective and frequently applied anticoagulants in clinical settings. They bind to antithrombotic proteins, including antithrombin (with the shortest effective unit being a pentasaccharide, which is used in fondaparinux) and heparin cofactor II (with the shortest effective unit being a hexasaccharide), inducing conformational changes that multiply their inhibitory effect. Antithrombin deactivates both thrombin and factor Xa when bound with heparin, whereas heparin cofactor II exclusively inhibits thrombin (48). This anticoagulant effect can be reversed by chemokines, which tightly bind heparin. For instance, CXCL4, a chemokine found in high abundance in blood plasma, can serve as an effective neutralizer of heparin (49, 50). Intravenous infusion of 5mg/kg CXCL4 fully reversed the anticoagulant activity of heparin in a small clinical trial in patients after cardiac surgery without any complications (51). Whether other abundant chemokines, such as CXCL7, would also result in heparin neutralization is, to our knowledge, unknown. Fluorescent probes enable the quantification of endogenous circulating GAG without differentiating the species or functional activity (52). In healthy human plasma, the concentration of circulating GAGs is in the range of 60 ng/ml. However, patients with pulmonary embolism, cancer, or liver disease may exhibit GAG levels exceeding 2 µg/ml (53).

### 2.2 Interactions between CXCL4 and anticoagulant and coagulant proteins

The use of homozygous and heterozygous knockouts as well as overexpressing knock-in mice, which either have a complete absence of CXCL4 or have reduced or increased CXCL4 levels, mimics the *in vitro* experiments in which a bell-shaped relationship between CXCL4 levels and prothrombotic function was observed (54). Compared with wild-type animals, both mice with increased and decreased CXCL4 expression showed a defect in arterial thrombosis formation (54). Despite full anticoagulation, infusion of heparin paradoxically enhanced thrombus formation in high CXCL4 mice, but potentiated the antithrombotic effect in low CXCL4 mice possibly by generating charge dependent attraction (54).

Another aspect of how CXCL4 affects the hemostatic system is by binding to proteins of the coagulation system that are simultaneously anti-inflammatory and fibrinolytic, such as activated protein C (APC), which can result in opposing effects. In humans, complete deficiency of APC, as in homozygous deficiency, results in microvascular thrombosis and hemorrhagic skin necrosis in infancy, whereas heterozygosity increases the risk of venous thrombosis in adults. Protein C is a vitamin K-dependent glycoprotein that is synthesized by the liver. After activation by thrombomodulin-chondroitinsulfate and its cofactor protein S, APC inactivates coagulation factors Va and VIIIa through proteolysis, resulting in reduced generation of thrombin, inhibition of thrombosis, and increased bleeding risk. CXCL4 has two effects on APC regulation. On one hand, CXCL4 enhances the thrombin/thrombomodulin dependent generation of APC on the endothelial surface and *in vitro* by interacting with the Gla domain of protein C and with chondroitinsulfate bound to thrombomodulin. This relationship does not follow a linear trend, as indicated by the bell-shaped curve, suggesting an optimal level for CXCL4 (55–57). This can be modulated by the presence of histones where low histone and high CXCL4 levels enhance APC formation and, in these models, high APC levels are associated with improved mouse survival (57). Extracellular histones activate platelets and induce thrombosis through a complement dependent mechanism. Mice deficient in complement component 5 (C5) are protected from histone-induced coagulopathy and lethal thrombosis (58). Platelets lacking C5R1 cannot be activated with C5 and therefore release less CXCL4. This mechanism has been shown to be important for angiogenesis but may also have implications for thrombosis (59).

By contrast, CXCL4 might foster blood clotting by impeding protein S’s augmenting effect on the anticoagulant function of APC through its interaction with APC’s Gla domain (60). Enhancing APC generation while simultaneously blocking its anticoagulant properties only makes sense if APC has multiple functional effects. Indeed, through its Gla domain, APC/Protein C binds to EPCR and PAR-1 on endothelial cells, thereby conferring cytoprotective effects. In this respect, it is noteworthy that CXCL4 did not block these cytoprotective functions of APC (60). It is possible that CXCL4 serves as a natural antagonist, blocking the risk of bleeding inherent to APC without jeopardizing protective functions. This is important under conditions of immunothrombosis/thromboinflammation, induced by activated platelets, when neutrophils expel web-like complexes of DNA, histones, chemokine and other mediators, a process termed NETosis (61, 62). Under these conditions, histones and CXCL4 additively modulate the rate of aPC formation. By inhibiting neutrophil EPCR, PAR3, and MAC-1, APC decreases NETosis. Consequently, in situations that result in platelet activation, this mechanism could serve as a crucial regulatory factor (63).

The role of CXCL4 in thrombosis and hemostasis is important but complex as it is involved in several systems regulating and keeping the balance in thrombosis and hemostasis by a corridor of concentrations that enhance platelet activation, whereas high and low concentrations will prevent this. Monitoring and adjusting blood concentrations would be required to therapeutically set an optimal balance between bleeding and thrombosis. Therefore, CXCL4 appears to be not an easy controllable ideal target.

### 2.3 CXCL7 variants bind and activate factor X

The other most abundant platelet chemokine CXCL7 (the gene is pro-platelet basic protein, PPBP), is stored as a full-length variant PBP consisting of residues 1-94, which become N-terminal truncated upon release to form CTAPIII (9-94) > β-TG (13-94) > NAP-2 (21-94) (64). The isoforms β-TG and CTAP III but not NAP-2 bind and activate coagulation factor X, thereby bypassing factor VIII and IX to support thrombin generation induced by the contact pathway, which may have relevance for hemophilia A and B (65). This effect was specific for CXCL7 as CXCL4 did not promote thrombin generation.

### 2.4 VWF forms thrombogenic complexes with CXCL4

Von Willebrand Factor (VWF) strings that form after endothelial injury under flow become decorated with CXCL4 (66). VWF is an anionic multidomain glycoprotein that forms multimeric end-to-end chains (concatemers) to achieve hemostasis after injury, but it can also provoke thrombosis. It is stored in endothelial Weibel-Palade bodies and in the α-granules of platelets, where it is folded into compact rods containing several thousand monomers (67, 68). Upon release, certain flow conditions unleash it into an elongated shape that exposes binding sites and enables it to function as a shear force sensor (67, 69). VWF binds factor VIII through its D modules and its C modules contain RGD motifs that recognize activated GPIIb/IIIa. Collagen binds to the A1 and A3 domains, platelet GP1b to the A1 domain, and the protease ADAMTS13 cleaves the A2 domain. ADAMTS13 regulates thrombosis and hemostasis by diminishing the size of the active VWF multimers and is, therefore, a critical antithrombotic molecule. Deficiency of ADAMTS13 leads to acquired thrombotic thrombocytopenic purpura (TTP). Consequently, it is important to note that CXCL4 binds to the A2 domain of VWF, thereby preventing its degradation (70). Moreover, circulating complexes of VWF and CXCL4 have been identified in patients with thrombocytopenic purpura, suggesting that CXCL4 contributes to microvascular thrombosis by inhibiting ADMTS13 (70). These circulating CXCL4•VWF complexes seem to mimic CXCL4•heparin complexes, as pathogenic antibodies isolated from the blood of HIT patients were also shown to recognize CXCL4•VWF (66).

## 3 Chemokines and platelets

### 3.1 Charge-driven effects of chemokines on platelets

CXCL4, along with proplatelet basic protein (PPBP), the full-length form of CXCL7, are signature chemokines of platelets. They are the most abundant circulating chemokines in the blood. With an isoelectric point (pI) of 8.8, mature CXCL4 is positively charged at physiological pH. This is thought to account for the contrasting effects of low and high CXCL4 levels on thrombosis investigated using transgenic mice either overexpressing human CXCL4 or being deficient for CXCL4 (54). CXCL7 is released as a proteolytic variant β-TG, which has an overall neutral charge with a pI of 6.9. The charge model proposes that the distance between platelets varies dependent on the extent of surface deposition of CXCL4. In the case of high or low CXCL4 levels, platelets are charged uniformly positive or negative, resulting in repulsive forces, whereas a moderate deposition level would allow attraction through mixed positive and negative charges (71).

### 3.2 Chemokine-chemokine receptor-mediated platelet activation

Of the 18 typical chemokine receptors that are coupled to G-proteins, specifically CCR1-10, CXCR1-CXCR6, XCR1, and CX3CR1 and the four chemokine receptors, ACKR1-4, that are considered atypical as they do not signal through G-proteins, RNA sequencing identified transcripts of all of them in human and mouse platelets, some only in traces and some at robust levels (**Table 2**).

**Table 2:** Expression of chemokine receptors by human and mouse platelets.

Chemokine receptor	Human, transcripts FKPM (72)	Human, transcripts mean RPKM (73)	Mouse, transcripts, mean RPKM (73)	Human platelet proteomes	FC, WB, qPCR	Functional expression
**CXCR4**	4.59	15.05	0.71	(72, 74–76)	(77)	(78)
**CCR4**	4.24	4.48	0.00	(72, 74, 75)		(78)
**CXCR2**	3.35	0.59	0.00			
**ACKR2 (D6, CCBP2)**	2.60	0.31	0.11	na		
**CX3CR1**	2.41	1.09	0.03	na		
**CXCR1**	2.07	0.01	0.00			(79)
**CCR5**	1.08	nd	0.00	na		
**CCR1**	1.03	0.45	0.00	na		(78)
**CCR7**	0.98	0.06	0.06			
**CXCR3**	0.75	0.11	0.00			
**CXCR5**	0.72	0.08	0.00	(80)		
**ACKR1 (DARC)**	0.69	0.07	0.15			
**CCR2**	0.59	0.44	0.00		(81)	(81)
**CCR3**	0.49	0.03	0.24			(78)
**ACKR3 (CXCR7)**	0.39	0.10	0.03			(82, 83)
**XCR1**	0.27	0.00	0.02			
**CCR6**	0.16	0.05	0.00			
**CCR9**	0.09	0.01	0.00			
**CXCR6**	0.01	0.02	0.00		(84)	(84)
**CCR10**	0.01	0.05	0.00	(80)		
**CCR8**	0.01	0.00	0.00			

The receptors are sorted according to the expression level of human platelets measured by transcript level according to the blueprint database (log2fpkm) (72). For comparison reasons human and mouse rpkm from earlier data are listed (73). Few chemokine receptors, most consistently human CXCR4 and CCR4 have been detected by proteomics, but no murine chemokine receptors. FC, flow cytometry; WB, Western Blot; qPCR, quantitative PCR.

Proteomics of human platelets (**Table 2**) picked up only CXCR4 and CCR4, the two receptors with the highest mRNA copy numbers suggesting that omics of platelets are unbiased but not completely specific or sensitive (72). Transcripts of CCR4 are missing in mice. Therefore, experiments exploring chemokines individually by functional and antibody-based tests are valuable. Presumably, those receptors with very low transcript copies will have no functional relevant protein expression, but drawing a cut-off line for relevant or irrelevant levels of transcripts is difficult.

Some of these chemokines have been used in platelet activation, aggregation, and signaling experiments to determine their biological function.

In an effort to comprehend the regulation of circulating platelet count, Gewirtz and colleagues searched and uncovered transcripts of CXCR1 and CXCR2 within human platelets and megakaryocytes. These receptors were responsive to CXCL7, which is one of the most highly expressed chemokines in platelets. They indeed found inhibition of megakaryocytopoiesis, suggesting a negative feedback loop through CXCR1 or CXCR2, although so far evidence of platelet activation by functional CXCR1/2 agonists CXCL8 or CXCL7 is elusive (79, 85). Later, Clemetson and colleagues (2000) revealed that CCL3, CCL4, CCL5, CCL11, CCL17, CCL22, and CXCL12 exert effects on aggregation and signaling in washed human platelets. These findings align with the expression of CCR1, CCR3, CCR4, and CXCR4 receptor mRNA or protein detected by the authors (78). Effects of CCL2 on platelet activation were judged ambiguous because CCR2 was not detectable, and the effects of CCL5 were not attributed to CCR5 because of lack of detectable expression and because CCR1 and CCR3 were sufficient to explain the effects (78). To date, CCL2’s effects on platelet activation have been reproduced, inhibited by CCR2 antagonists, and appear to be effective *in vivo*, as CCL2 knockout mice show mildly impaired hemostasis and arterial thrombosis (81).

CXCR4 is robustly expressed by platelets and megakaryocytes (**Table 2**). First studies identifying functional CXCR4 expressed by the MK/platelet lineage showed that the CXCL12-CXCR4 axis is a negative regulator of megakaryopoiesis/thrombopoiesis (77). Analyzing platelet function using washed platelets can deteriorate the sensitivity of an assay because methodical differences in washing procedures and analysis buffers can mask the effects of mediators with low potency. At least this is one explanation for why CXCL12-induced aggregation of washed platelets has only been observed as a synergistic increase in platelet response to a coagonist. In contrast, platelet aggregation was observed in platelet-rich plasma or whole blood when CXCL12 was used as the only agent (30, 31, 78, 86–90). In the flow chamber, CXCL12-induced thrombus formation mainly affects the 3D thrombus build-up while initial platelet deposition at the surface is only mildly reduced (31). Platelets are a relevant source of CXCL12, and using CXCR4 inhibitors and platelet-specific CXCL12- and CXCR4 knockout mice has clarified that an endogenous feedback loop exists (91). Solubilized plaque material mimicking atherosclerotic plaque rupture activates platelets that release CXCL12, thereby contributing to arterial thrombosis in a mouse model (31, 87). The activation of platelets through the CXCL12-CXCR4 axis leads to signals in the Gi-, PI3Kβ, BTK-, AKt-, and p38MAPK pathways that result in calcium influx, thromboxane and granule release and activation of GPIIb/IIIa (87, 89, 90). The phosphorylation of BTK after CXCR4 activation and the observation that platelet activation induced by CXCL12 can be eliminated with BTK inhibitors, suggests that BTK acts as a critical signaling molecule downstream of platelet chemokine receptors in addition to the recognized ITAM-coupled platelet immune receptors (31, 92, 93).

Beyond the platelet-dependent role of CXCL12 in atherothrombosis, platelets and likely also CXCL12 play an essential role in venous thrombosis (94). At the translational level, CXCL12 activity is regulated by microRNAs (miRs) and posttranslational by proteolytic processing with impact on venous thrombosis. Several circulating miRs including miR 103a-3p have been described to be biomarkers in patients with VTE. Circulating levels of miRNA 103a-3p are decreased in individuals with deep vein thrombosis, and CXCL12 is a target of miRNA 103a-3p, which may explain the observed increase in plasma CXCL12 levels in these patients (95). Appropriately, the overexpression of miR 103a-3p resulted in a reduction of CXCL12 protein levels in the bloodstream and inhibited venous thrombosis in mice, thus providing further support of the concept (95).

CXCL12 exists in various forms of different lengths. Truncation of the first two N-terminal residues leads to CXCL12[24-88], which exhibits unique binding properties and reduced activation of CXCR4. Additional proteolytic processing renders CXCL12 inactive or transforms it into an antagonist (96). Dipeptidylpeptidase 4 (DPP4) is a widely distributed enzyme found on both epithelial and endothelial cells. It cleaves after the first two aminoterminal residues in the consensus sequence of CXCL12 (KP-VSL…), resulting in the production of CXCL12[24-88] but also cleaves and deactivates the incretin GLP-1 (96, 97). Therefore DPP4-inhibitors are commonly used drugs in type 2 diabetics. Blocking DPP4 may potentially increase active CXCL12 levels and augment thrombus formation, as suggested by retrospective data from the WHO spontaneous reporting database. However, this is speculative and not confirmed by a later meta-analysis that analyzed 5 large cardiovascular outcome trials and did not find any signal regarding DPP4-associated VTE (98, 99).

CXCL12 (SDF) is the only typical chemokine that binds to and activates CXCR4. In comparison, the atypical chemokine MIF is another non-cognate ligand displaying partial agonism, and CXCL14 is an additional ambiguous candidate, indicated to synergize with CXCL12 or potentially manifest effects by directly binding CXCR4 (100–104). In contrast to classical chemokines, MIF lacks an N-terminal cysteine motif, though the structure of the MIF monomer bears a resemblance to that of a CXCL8 dimer. MIF is stored and produced by platelets, but the kinetics of secretion are slower than those of chemokines (105). Although MIF can bind to chemokine receptor complexes on platelets, it does not activate platelets. Rather, it keeps them in a resting state and attenuates *in vitro* thrombus formation at high shear flow on a collagen surface (83). In humans, MIF interacts with CXCL4L1 (also known as PF4V1 or PF4alt), a non-allelic variant of CXCL4, to produce prothrombotic and proinflammatory MIF-CXCL4L1 heterocomplexes. This mechanism is absent in mice, which lack the orthologue for CXCL4L1 (PF4V1) (106).

**Figure 2:**
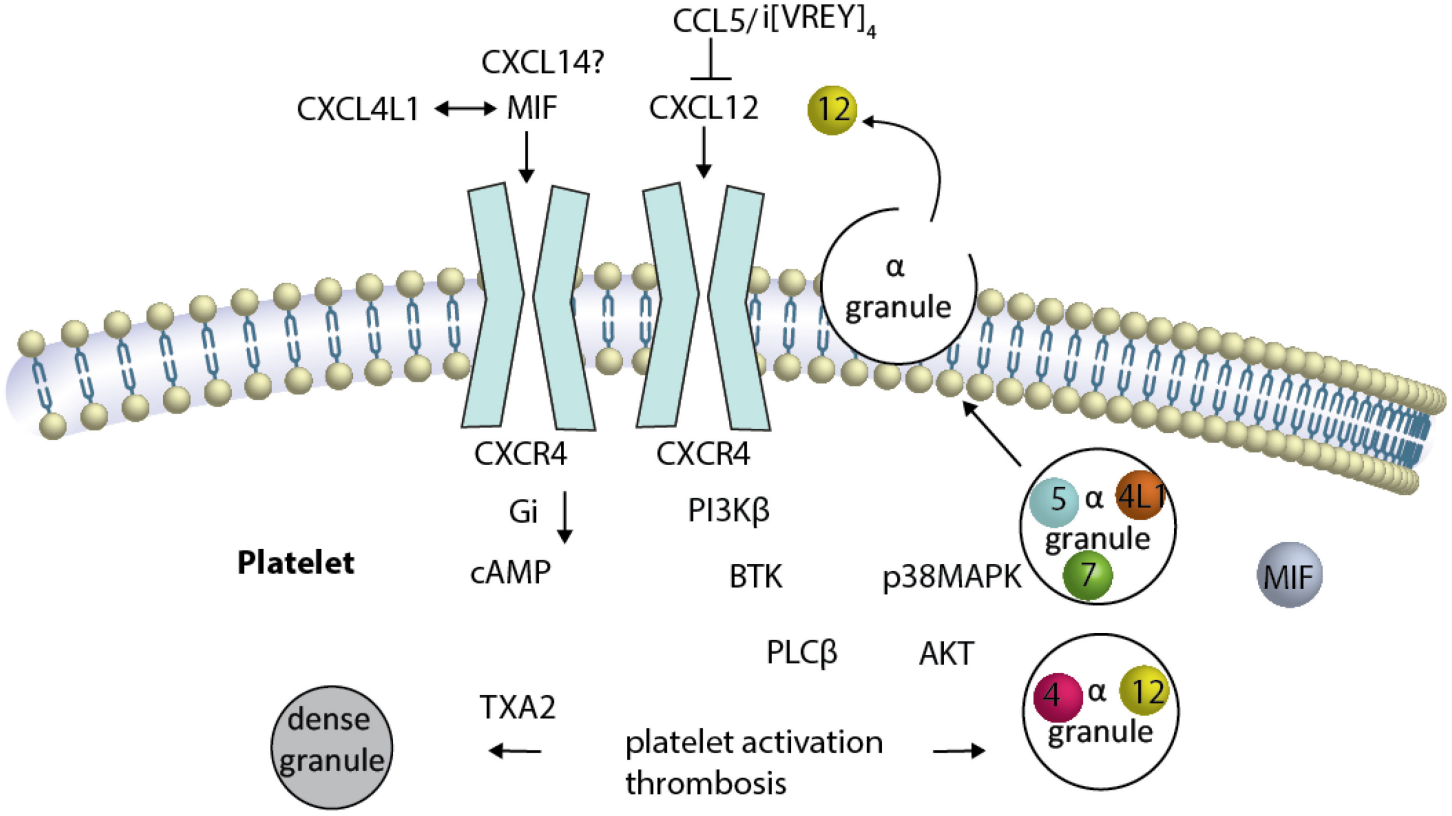
Platelet CXCR4 is a driver of platelet activation, and thrombosis CXCR4 is illustrated as a dimer (although this has not been formally shown in platelets). Activation of and Gαi-coupled CXCR4 by CXCL12 induces reduction of elevated cAMP. Several signaling pathways have been described to be activated either separately or by crosstalk of the signaling components, resulting in platelet activation and thrombosis. The sequence of events leading from CXCR4 activation to BTK-phosphorylation is unknown. CXCL12-induced platelet aggregation can be blunted by the inhibition of PI3K, BTK … TXA2. The release of alpha granules containing CXCL12, CXCL4, CCL5, CXCL7 (spheres) and other chemokines (not MIF), as well as the TXA2-mediated release of dense granules, results in platelet activation loops. The newly released CXCL12 activates CXCR4 in a positive-forward loop.

Furthermore, inhibition of CXCL12-induced platelet activation has been shown by the presence of CCL5, which can also be released by platelets (107, 108). CCL5 inhibits CXCL12 in a non-competitive manner (107), likely due to formation of a heterodimer (109). The carboxyterminal α-helix of CCL5 has been identified as an important region for this complex formation, and the generation of a bundled peptide of this sequence results in a peptide compound that binds to CXCL12 and CXCR4 at the same time (**Figure 2**), thereby partially preventing CXCR4-activation (31).

High levels of CXCL12 and CXCR4 on the surface of platelets are biomarkers that predict increased cardiovascular mortality due to arterial thrombosis. However, the functional significance remains unclear. It is possible that these biomarkers reflect a prothrombotic state resulting from increased platelet reactivity (110, 111).

Endowed with a platelet inhibiting function, ACKR3 (CXCR7) has been stained on the surface of platelets as a receptor for MIF, CXCL12, and CXCL11 and interacts with CXCR4 forming heterocomplexes (112–114). Stimulation of platelets with CXCL12 leads to internalization of CXCR4 but increased surface exposure of ACKR3 through crosstalk with CXCR4 (82). Although CXCR7 does not affect Gαi-signaling directly, interacting with CXCR4 inhibits CXCR4-signaling through Gαi (113). Whether and how CXCR4/CXCR7 assemble into heteromeric complexes in primary cells and platelets has yet to be established.

Finally, the CXCL16-CXCR6 axis has been described as instrumental for platelet activation in humans and mice but requires a co-agonist like ADP (84, 115). Platelet-CXCL16 levels are an independent predictor for all-cause of mortality in patients with stable and unstable CAD (116).

### 3.3 Platelet thrombi direct leukocytes into the vessel wall using CXCL7 gradients

Platelet-derived chemokines have the potential to establish gradients within platelet thrombi, with elevated concentrations bordering the vascular wall and lower concentrations on the luminal side (117). Neutrophil-platelet thrombus interactions were investigated using a CXCL7 knockout mouse model. The results indicated that neutrophils can bind to, invade, and transmigrate through platelet thrombi in a CXCL7-dependent manner (117). Whether this constitutes a regulatory aspect of a healing process or a harmful tissue inflammation and its effect on thrombus size and resolution are yet to be determined.

## 4 Leukocyte subsets of the innate and adaptive immune system are recruited to early thrombosis and affect thromboinflammation by complex formation in NETs, HIT and VITT

CXCL4 and, to a lesser extent, other chemokines (e.g., CXCL8) serve as antigens for pathogenic antibodies released by a limited number of B cell/plasma cell clones. This leads to the formation of immune complexes that activate platelets and occlusive platelet thrombi that can form in various apparently healthy vascular beds such as splanchnic veins, sinus veins and arteries (118–120). Entities of immune complex thrombosis comprise HIT which necessitates heparin-chemokine complexes, autoimmune HIT without therapeutic heparin as a trigger, and VITT. The latter came into view at the beginning of the COVID-19 vaccination campaign with adenovirus vaccines. The biological activity of the chemokine appears to be irrelevant in these thrombotic entities. The chemokine serves as a structural link between the antibodies and another component. In the case of HIT, this component may be heparin, while in the case of aHIT and VITT, it may be an unknown polyanionic molecule, such as non-heparin GAG, DNA, polyphosphate, or VWF. Presumably, the exceptionally high blood concentration, the tendency to form tetramers, and a very high affinity for heparin or another polyanion are the conditions that set CXCL4 above other chemokines in this regard. HIT, aHIT, and VITT may have varying polyanionic components that trigger the immune response, but the signaling pathways are highly similar. For the mechanism of platelet activation, the formation of antibody complexes is essential because the receptors that recognize these antibodies on platelets, particularly FcγRIIa (CD32A), require clustering to activate and initiate downstream signaling through the PI3K, BTK, and PLC. In this respect, it is noteworthy and therapeutically relevant that Btk inhibitors demonstrated encouraging outcomes in averting platelet activation through HIT and VITT sera (121–123). For more information on the pathogenesis, diagnostic methods, and therapeutic approaches to immune complex thrombosis, please refer to published reviews (124–126).

Leukocytes, particularly neutrophils, participate in immunothrombosis by releasing extracellular DNA traps (in the case of neutrophils termed NETs, NETosis) a tangle of DNA, histones citrullinated by PAD4, and antimicrobial granule proteins (**Figure 1**) (62). NETs are detectable in human ischemic stroke thrombi and in human coronaries and their presence has prognostic impact (127, 128). Blocking neutrophil rolling and adhesion during flow restriction in the vena cava has been demonstrated to decrease thrombosis rates and sizes by examining different leukocyte recruitment receptor knockouts. CXCR2, PSGL1, and the myeloid integrin CD11b (αMβ2), but not L-selectin knockouts, exhibit partial protection against venous thrombosis (129). See **Table 3** for chemokine receptor expression in monocytes and neutrophils. Neutrophil rolling, facilitated by the P-selectin-PSGL-1-axis, is an essential initial step towards adhesion. During thrombosis, CXCL1-CXCR2 signaling and signals transmitted by the interaction of P-selectin-PSGL-1 not only cooperatively induce adhesion but also NETosis. The combination of both activation pathways can further enhance this process (129).

Table 3Gene expression of chemokine receptors of leukocyte subsets from human blood.

**Table:** 

Leukocyte subset	No. of cells	Readout	CCR1	CCR2	CCR3	CCR4	CCR5	CCR6	CCR7	CCR8	CCR9	CCR10
Classical monocyte	7210	log2cpm	1.6 ± 1.8	2.9 ± 3.0	0.0 ± 0.3	0.0 ± 0.2	0.0 ± 0.4	0.0 ± 0.0	0.0 ± 0.1	0.0 ± 0.2	0.0 ± 0.1	0.0 ± 0.5
		% pos.	50.5	56.1	0.1	0.4	0.7	0.0	0.3	0.0	0.0	0.9
Non-classical monocyte	8	log2cpm	1.2 ± 1.3	0.0 ± 0.0	0.0 ± 0.0	0.0 ± 0.0	0.0 ± 0.0	0.0 ± 0.0	0.0 ± 0.0	0.0 ± 0.0	0.0 ± 0.0	0.0 ± 0.0
		% pos.	50.0	0.0	0.0	0.0	0.0	0.0	0.0	0.0	0.0	0.0
Monocyte	8971	log2cpm	1.2 ± 1.8	1.9 ± 2.7	0.0 ± 0.2	0.0 ± 0.2	0.1 ± 0.7	0.0 ± 0.0	0.0 ± 0.0	0.0 ± 0.0	0.0 ± 0.1	0.1 ± 0.6
		% pos.	36.9	38.1	0.9	1.3	3.4	0.9	0.9	0.9	0.9	2.0
Neutrophil	8454	log2cpm	0.3 ± 1.2	0.0 ± 0.2	0.0 ± 0.4	0.0 ± 0.4	0.0 ± 0.0	0.0 ± 0.2	0.0 ± 0.0	0.0 ± 0.0	0.0 ± 0.0	0.0 ± 0.2
		% pos.	5.8	0.2	0.1	0.3	0.0	0.0	0.0	0.0	0.0	0.0

**Table:** 

Leukocyte subset	No. of cells	Readout	ACKR1	ACKR2	ACKR3	ACKR4	CX3CR1	XCR1	CXCR1	CXCR2	CXCR3	CXCR4	CXCR5	CXCR6	CXCR8
Classical monocyte	7210	log2cpm	0.0 ± 0.0	0.0 ± 0.2	0.0 ± 0.2	0.0 ± 0.2	0.1 ± 0.8	0.0 ± 0.2	0.3 ± 1.1	0.3 ± 1.0	0.0 ± 0.1	0.5 ± 0.5	0.0 ± 0.1	0.0 ± 0.0	0.0 ± 0.2
		% pos.	0.0	0.1	1.4	0.2	3.8	0.0	7.0	13.1	0.1	60.8	0.2	0.0	12.5
Non-classical monocyte	8	log2cpm	0.0 ± 0.0	0.0 ± 0.0	1.2 ± 1.4	0.0 ± 0.0	0.8 ± 0.9	0.0 ± 0.0	0.0 ± 0.0	0.0 ± 0.0	0.0 ± 0.0	1.1 ± 0.3	0.0 ± 0.0	0.0 ± 0.0	0.0 ± 0.0
		% pos.	0.0	0.0	62.5	0.0	25.0	0.0	0.0	0.0	0.0	100.0	0.0	0.0	0.0
Monocyte	8971	log2cpm	0.0 ± 0.0	0.0 ± 0.2	0.0 ± 0.2	0.0 ± 0.3	1.0 ± 2.2	0.0 ± 0.2	0.1 ± 0.4	0.1 ± 0.5	0.0 ± 0.0	0.4 ± 0.4	0.0 ± 0.0	0.0 ± 0.0	0.4 ± 1.4
		% pos.	0.9	1.0	3.6	1.0	21.1	0.9	2.9	6.4	0.9	50.5	0.9	0.9	8.9
Neutrophil	8454	log2cpm	0.0 ± 0.0	0.0 ± 0.1	0.0 ± 0.0	0.0 ± 0.3	0.1 ± 0.7	0.0 ± 0.2	3.8 ± 3.7	4.6 ± 2.7	0.0 ± 0.0	0.7 ± 0.7	0.0 ± 0.0	0.0 ± 0.0	0.0 ± 0.3
		% pos.	0.0	0.0	0.0	0.1	0.9	0.0	55.0	79.4	0.0	54.9	0.0	0.0	0.1

Gene expression of chemokine receptors of monocytes and neutrophils from human blood. Data are derived from the Tabula sapiens. Indicated are expression levels as mean ± SEM of log2cpm and percentage positive cells. Note that data from only 8 non-classical monocytes may not be representative. The color intensity highlights and corresponds to the expression level. ACKR3=CXCR7.

Activated platelets and several potent inflammatory mediators in sepsis and at high concentrations CXCL8 (IL-8) can activate neutrophils to release NETs (61, 130, 131). In COVID-19, the CXCL8-CXCR1/-2 axis plays an important role through an autocrine forward loop when neutrophils are simultaneously mobilized and activated by CXCL8 and contribute to microvascular thrombosis of the pulmonary vasculature by NETosis (132). This is an example of how NETs arise in immunothrombosis as a defense mechanism in infections and sepsis to protect the organism by trapping infectious pathogens (2). By incorporating red blood cells, NETs contribute to the formation of red thrombi and are major drivers of deep vein thrombosis. NETs are found in HIT, also in VITT models, and arterial thrombosis (38, 62, 133, 134). Therefore, they are important for sterile thromboinflammation in all vascular beds. Chemokines are at play because they bind to DNA and are found in NETs. Platelet chemokines, which are released during thrombosis, rapidly come into contact with NETs. CXCL4 specifically binds to cell-free DNA under flow and is integrated into NETs, making them more compact and resistant to nuclease degradation (133). CXCL4 organizes double-stranded self-DNA and bacterial DNA into liquid-crystalline supramolecular complexes (135). This is directly relevant to NETosis, thereby depleting free bacteria and improving outcome in murine models of sepsis (136). However, at the same time, this effect may enhance an ongoing thrombotic process as well. DNA•CXCL4 complexes facilitate cellular internalization, TLR9 binding, activation, and IFN-α release and therefore are thought to be more immunogenic and might brake tolerance to self-DNA, which has, in turn, implications for immunological thromboses (HIT, VITT) (135). This principle was initially demonstrated for CXCL4 and applies to some other chemokines as well. Possibly the heteromer formation of CCL5•CXCL4 also plays a role because inhibition of CCL5•CXCL4 preserves heart function after myocardial infarction by attenuating leukocyte recruitment and NETosis (137).

In addition to CXCL4, CXCL10, CXCL12 and CCL5 amplify TLR9-IFN-I responses that require their binding to DNA. Not all chemokines bind DNA. For instance CXCL7/Nap-2, CXCL9 and other chemokines have no IFN-I inducing effect, which excludes unspecific charge driven chemokine•DNA effects (138). In addition, and similar to CXCL4, CXCL10 binds bacterial DNA and enhances an adjuvant immune response mouse wound injury (139). CXCL10 and CXCL8 levels in the blood of septic patients and CXCL2 in mouse models (no CXCL8 gene in mice) correlate positively with septic NET formation, and simultaneous blockade of CXCR1 and CXCR2 with the allosteric inhibitor reparixin prevent NET formation *in vitro* and *in vivo* and improved survival in sepsis models (140). However, binding studies of these other chemokine DNA complexes, except for CXCL10, do not yet exist so that detailed structural insights on the epitopes and contact sites are missing. That heparin partly inhibits the release of interferon by several chemokines complexed with DNA implies that there are common binding sites that overlap (138).

Besides neutrophils, monocytes cover dysfunctional prothrombotic endothelium at the early stage of venous thrombosis and expose tissue factor (TF), a transmembrane receptor initiating the extrinsic pathway of coagulation (**Figure 1**) (38). Typically, TF is selectively expressed by perivascular and epithelial cells. However, monocytes are able to transiently express TF, when induced by LPS-mediated TLR activation or after surgery (141). Preferentially mouse Ly6C^high^, inflammatory, monocytes seem to express tissue factor (TF), at least after LPS treatment (142). They express highly CCR2. In addition, other leukocyte subsets, including dendritic cells, plasmacytoid dendritic cells, and NK cells, as well as inflamed endothelial cells, express CCR2 (143). Knocking out CCR2 results in sequestration of monocytes in the bone marrow and severe reduction in circulating monocyte count (143, 144). Reducing circulating monocytes through various methods, such as anti-CCR2, anti-CCL2, clodronate depletion, CCR2 knockout, or converting inflammatory Ly6C^high^ monocytes to Ly6C^low^ monocytes using the Nur77 agonist Cytosporone B (Csnp), leads to slower thrombus growth and faster resolution (142, 145). Reducing monocytes to limit thrombosis and thrombus resolution seems counterintuitive since CCL2 promotes thrombus-invading monocytes to accelerate thrombolysis (16, 146). However, the CsnB-application after four days of thrombus growth showed indeed faster resolution of the venous thrombus (142). One speculative explanation would be that not inflammatory but rather patrolling Ly6C^low^ monocytes are the active force behind thrombus resolution or off-target effects of Csnp could be accountable.

The expression of CCR2 and CCL2 in monocytes and endothelial cells are both regulated by the vitamin K-dependent, Gla-domain containing plasmatic factor Gas6 which is the ligand for the myeloid receptor tyrosine kinases MerTK. Gas6 knockout leads to decreased expression of both the CCL2 chemokine and its corresponding receptor, CCR2. In venous thrombosis induced either in the vena cava through flow restriction or by FeCl3 injury, Gas6 deficiency results in smaller thrombi containing fewer monocytes but normal levels of neutrophils. This suggests that Gas6 specifically promotes the recruitment of inflammatory monocytes by regulating both CCR2 and CCL2 (145).

Chemokines promote venous thrombosis by recruiting monocytes that contain tissue factor via the CCL2-CCR2 pathway. Simultaneously, they recruit and activate neutrophils that produce (NETs) via the CXCL1-CXCR2 axis. Targeting leukocyte adhesion by blocking rolling or the conversion from rolling to adhesion by antibodies against P-selectin or PSGL1 by blocking chemokines or their receptors or blocking NETosis are conceivable strategies in prevention and treatment of thrombosis. Advantages of anti-inflammatory therapeutics over anticoagulants could be that the sequelae of thrombosis would be targeted, i.e. damaged valves and the postthrombotic syndrome could benefit from an anti-recruitment therapy. Possible downsides are that inflammation is supposed to be a regulatory requirement for proper healing and hemostasis. It is yet unclear to what extent anti-inflammatory treatment is safe or if the intimate relationship between clotting and inflammation is a weakness in the system and a gateway to bleeding. Chemokines can also contribute to NETosis independently of any chemokine receptor by binding to DNA in NETs and affecting their structure. This process appears to be specific to a limited number of chemokines, despite the lack of systematic profiling regarding their affinity and the ambiguous nature of their binding sites. Other questions need to be answered in the future, too. Are chemokines that are entangled in NETs still able to bind to their receptors and functionally active? What occurs to the oligomeric nature of chemokines in these structures? Are chemokines present in NETs engaging in competition with other DNA-binding proteins, such as histones and myeloperoxidase (MPO), and subsequently releasing them?

**Figure 3:**
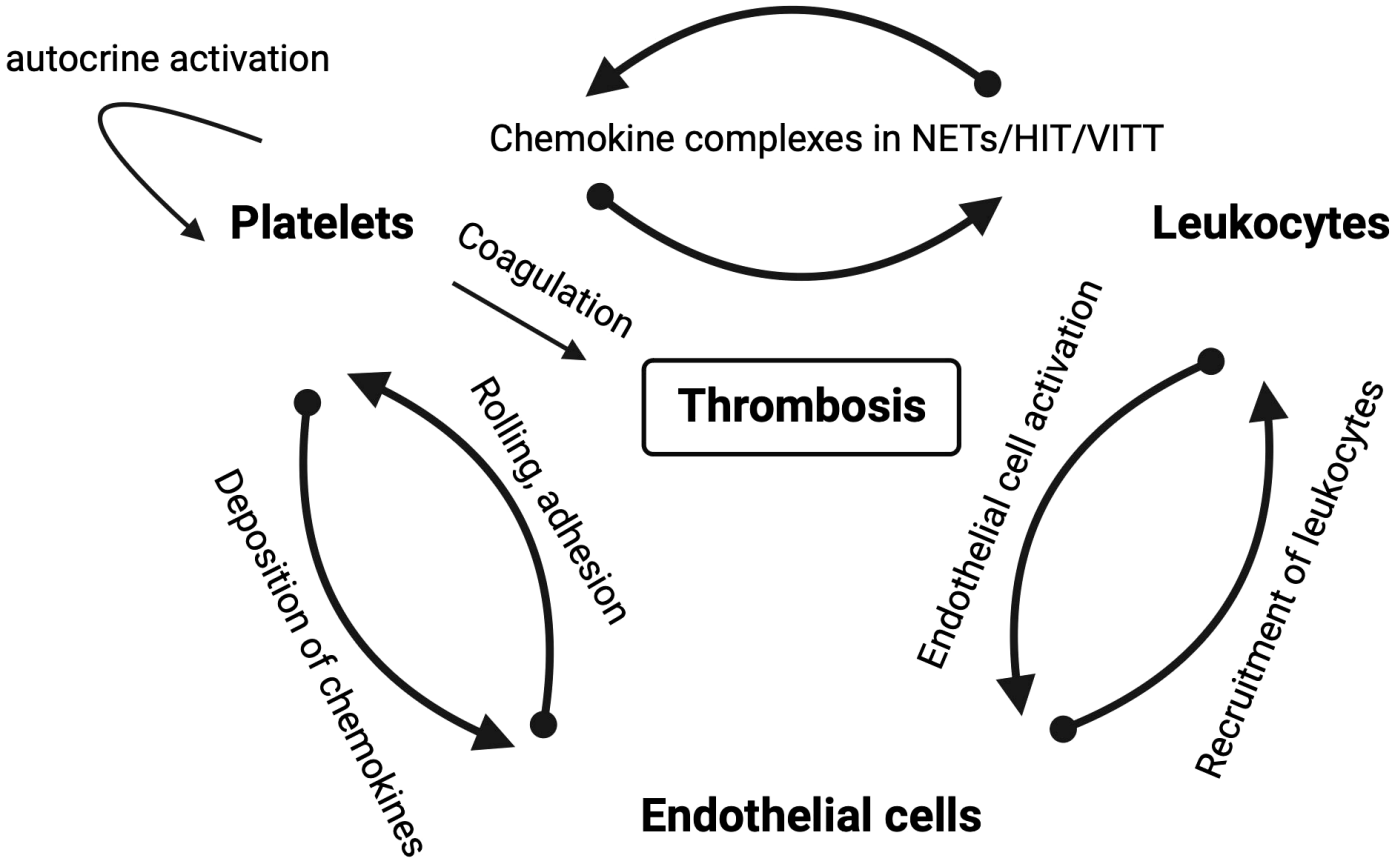
Schematic overview of mutual interactions of platelets, leukocytes and endothelial cells in thrombotic diseases in which chemokines play a role. Arrows depict the direction of the interaction labeled with the mode of interaction.

Chemokines and their receptors are potential targets in NETosis and, consequently, in immunothrombosis and thromboinflammation (**Figure 1**). However, inhibiting NETosis may have both positive and negative effects, and the timing and balance between inflammatory and thrombotic conditions are crucial for the success of any inhibitory therapy. Chemokines are effectors of mutual interactions of platelets, leukocytes and endothelial thereby driving thromboinflammation and immunothrombosis (**Figure 3**).

## 5 Chemokines, erythrocytes and ACKR1 in venous and arterial thrombosis

Traditionally, arterial thrombosis has been described as predominantly platelet-dependent due to the platelet-rich and erythrocyte-poor white morphology of thrombi. In contrast, venous thrombi show the opposite pattern and are characterized as red with low platelet and high erythrocyte content. Clinical data justify this concept because antiplatelet agents are proven superior over anticoagulation in preventing arterial thrombosis in myocardial infarction and atherosclerotic stroke. Thrombosis in the venous system, including deep vein thrombosis and atrial and auricular thrombus formation, can be treated and prevented more effectively with anticoagulants than with antiplatelet therapies. While this picture of arterial white platelet and red venous thrombi may hold some truth, it is known to be at least too simplistic and in angioscopic studies, coronary thrombi can be red or white with STEMI thrombi being more often red and those associated with unstable angina being more often white and antiplatelets have some efficacy in venous thrombosis (147, 148). Red blood cells in arterial thrombosis deserve attention. Their role has been demonstrated in FeCl3-induced endothelial injury, where the initial response came not from platelets, but from RBCs (149, 150).

Many chemokines and atypical chemokines, including MIF, may not be synthesized by RBCs or their precursors. Yet, they are impressively present at the protein level and represent a large reservoir in the blood (151, 152). ACKR1 plays an essential role in this respect. In addition to endothelial cells, ACKR1 is expressed at high levels by erythrocytes, except in a majority of West Africans or African Americans who carry a genetic polymorphism in the GATA-1 binding site that prevents ACKR1 expression on erythrocytes. ACKR1 binds to about 20 proinflammatory chemokines of the CC- and CXC-family such as CCL2, CCL5, CXCL5, and CXCL8 but not CXCL4, CXCL10 and CXCL12 (152–154). ACKR1 (gene Fy) possesses a common single nucleotide polymorphism in the extracellular N-terminal domain, which is important for chemokine binding. The major allele (FYB) translates at position 42 into aspartate, which is exchanged in the minor allele to a glycine (FYA). A GWAS study comparing several large cohorts found a strong association of this polymorphism with serum concentration of CCL2 and, to a lesser extent, with CCL5 and CXCL8 but not with CXCL10 that does not bind ACKR1. Notably, this observation was only accurate under coagulation conditions, as the correlation was solely observable in serum and not in plasma (155). This indicates that individuals who carry the FYB gene release higher levels of inflammatory chemokines while clotting due to an unknown factor, which may impact the development and resolution of thrombosis.

## 6 Conclusions

Inflammation and thrombosis are inextricably linked, and chemokines and their receptors are key regulators involved in several aspects of thromboinflammation and immunothrombosis by affecting circulating leukocyte numbers, leukocyte activation and recruitment during all stages of thrombosis and also by directly activating platelets and modulating the coagulation system. Although arterial thrombosis, as in HIT, as opposed to plaque rupture and atherothrombosis, microvascular thrombosis and venous thrombosis have different origins, there are many similarities in the progression of thrombosis. For example, shear gradients, NETosis, leukocyte recruitment, the contribution of red blood cells, activated endothelial cells, and activated platelets are relevant in each of these cases. However, we know that although anticoagulation and antiplatelet therapy (APT) have some efficacy in both arterial and venous thrombosis, anticoagulation is clearly preferred for venous thrombotic disease and APT for atherothrombosis whereas the treatment of immunocomplex thromboses is less standardized and depends on whether heparin is part of the pathology or not. Using the chemokine system as a target holds some promise but it needs to be tailored. Moreover, the knowledge we have gained is mostly from animal models of thrombosis and usually a comparison of thrombosis in distinct vascular beds and types of thrombosis is not available and a longer-term use and broader evaluation of effects on health are difficult to predict.

Most human chemokines also exist in mice and can bind and activate mouse chemokine receptors. However, transferability is not universally applicable, and some species differences have been identified. Differences in gene duplication may lead to different expression of CCL19, CCL22, and CCL27, which are the CCR7-ligands, between mice and humans. This disparity may complicate comparisons between the murine and human systems (156, 157). Furthermore, mice do not possess orthologs for human CXCL4L1, CXCL6, and CXCL8. Human CXCL1 and CXCL8 activate mouse CXCR1 upon binding, prompting the use of human chemokines for *in vivo* assays (158, 159). Another instance of interspecies variation lies in specific differences at the cellular level, exemplified by CCR4 (**Table 2**). While human platelets express this element, their mouse counterparts do not. Therefore, platelet activation via CCR4 by CCL17 should be operable only in humans. Mouse models are essential for investigating thrombosis *in vivo*, but the transferability of findings to the human system must be independently determined. In the translational process, clinical trials can provide valuable insight into the safety of utilizing chemokine therapeutics in other diseases. For instance, studies have investigated the prevention of diabetic nephropathy progression through the blocking of monocyte recruitment with small molecules, such as a specific CCR2 or dual CCR2/CCR5 antagonist (160, 161). Applying these antagonists to human subjects for either twelve or fifty-two weeks appears to be safe, with a notable decrease in circulating monocytes and a concomitant increase in CCL2 blood concentrations.

Targeting chemokines or their receptors may have an impact on circulating leukocyte numbers and potentially disrupt leukocyte homeostasis. Mobilization of leukocytes from the bone marrow is a finely tuned and temporally phased process that is amplified by inflammation as a trigger. The main cytokine axis in granulopoiesis is G-CSF/G-CSFR, which interacts with CXCR4 and CXCR2 to control the release of neutrophils from the bone marrow. The bone marrow CXCL12/CXCR4 axis restricts neutrophil mobilization. G-CSF downregulates the expression of CXCR4 on neutrophils and CXCL12 in bone marrow, which disrupts neutrophil retention (162). Similarly, certain CXCR4 antagonists result in neutrophil release whereas others such as the biased antagonist peptide i[VREY]_4_ do not (31). In contrast, the expression of CXCR2 by neutrophils leads to a rapid release from the bone marrow when CXCR2 ligands (CXCL8, CXCL1, CXCL2) are upregulated. Unexpectedly, G-CSF blocks CXCR2-signaling and regulates neutrophil mobilization negatively (163). Therefore, the cytokine G-CSF has opposing roles on neutrophil release depending on the phase by affecting more than on chemokine pathway. Monocytes are mobilized from the bone marrow into the circulation by CCL2 and CCL3 through monocytic CCR2 (164). Increased monocyte counts in the peripheral blood correlate with venous thrombosis in the mouse (142). It is unclear whether neutrophils will pave the way for monocytes or both accumulate simultaneously. As these leukocyte subsets express several chemokine receptors (**Table 3**) it could be necessary to block multiple receptors to achieve a meaningful effects. Blocking CXCL12 activity through small molecules inhibits CXCR4 completely, which inhibits platelet aggregation and arterial thrombosis, yet leads to leukocytosis whereas using biased or partial antagonists affect signaling differentially to preserve homeostatic functions and do not result in leukocytosis. When dealing with chemokine signaling, it is crucial to comprehend the points at which the distinct pathways diverge in order to target specific prothrombotic pathways for inhibition. Heparins are perhaps the oldest antithrombotics and chemokine scavengers that neutralize chemokine activity. However, due their unselective binding to virtual all chemokines and many other proteins their anti-chemokine role in thrombosis is difficult to dissect and engineering specific and orally available GAGs that do not affect coagulation will be difficult. Similarly, evasin peptides, which are multi-chemokine binders produced by ticks, have not yet undergone assessment for their potential role in thrombosis (165). It needs to be determined how targeting chemokines in thrombotic diseases will integrate into the current therapeutic strategies. Applying them earlier may have a greater impact on thrombosis, while resolution of the thrombus may be hindered at later timepoints. Potential adverse effects of effective chemokine inhibition may comprise bleeding and dysregulation of the immune system. Nevertheless, bleeding has not been described in any animal models utilizing anti-chemokine strategies. If combined with anticoagulants or antiplatelet therapy, however, there may be a collaborative antihemostatic effect.

## Author contributions

PvH: Funding acquisition, Supervision, Visualization, Writing – original draft, Writing – review and editing. JL: Visualization, Writing – review and editing.
